# Dosimetry in Micro-computed Tomography: a Review of the Measurement Methods, Impacts, and Characterization of the Quantum GX Imaging System

**DOI:** 10.1007/s11307-016-1026-x

**Published:** 2016-12-12

**Authors:** Jeffrey A. Meganck, Bob Liu

**Affiliations:** 1 Research and Development, Life Sciences Technology, PerkinElmer, 68 Elm Street, Hopkinton, MA 01748 USA; 2 Department of Radiology, Massachusetts General Hospital, Boston, MA USA

**Keywords:** X-ray micro-computed tomography, Half value layer (HVL), Radiation dose

## Abstract

**Purpose:**

X-ray micro-computed tomography (μCT) is a widely used imaging modality in preclinical research with applications in many areas including orthopedics, pulmonology, oncology, cardiology, and infectious disease. X-rays are a form of ionizing radiation and, therefore, can potentially induce damage and cause detrimental effects. Previous reviews have touched on these effects but have not comprehensively covered the possible implications on study results. Furthermore, interpreting data across these studies is difficult because there is no widely accepted dose characterization methodology for preclinical μCT. The purpose of this paper is to ensure *in vivo* μCT studies can be properly designed and the data can be appropriately interpreted.

**Procedures:**

Studies from the scientific literature that investigate the biological effects of radiation doses relevant to μCT were reviewed. The different dose measurement methodologies used in the peer-reviewed literature were also reviewed. The CT dose index 100 (CTDI_100_) was then measured on the Quantum GX μCT instrument. A low contrast phantom, a hydroxyapatite phantom, and a mouse were also imaged to provide examples of how the dose can affect image quality.

**Results:**

Data in the scientific literature indicate that scenarios exist where radiation doses used in μCT imaging are high enough to potentially bias experimental results. The significance of this effect may relate to the study outcome and tissue being imaged. CTDI_100_ is a reasonable metric to use for dose characterization in μCT. Dose rates in the Quantum GX vary based on the amount of material in the beam path and are a function of X-ray tube voltage. The CTDI_100_ in air for a Quantum GX can be as low as 5.1 mGy for a 50 kVp scan and 9.9 mGy for a 90 kVp scan. This dose is low enough to visualize bone both in a mouse image and in a hydroxyapatite phantom, but applications requiring higher resolution in a mouse or less noise in a low-contrast phantom benefit from longer scan times with increased dose.

**Conclusions:**

Dose management should be considered when designing μCT studies. Dose rates in the Quantum GX are compatible with longitudinal μCT imaging.

## Introduction

The field of medical imaging utilizes many different imaging and detection methodologies. Magnetic resonance imaging, ultrasound, and optical imaging do not use ionizing radiation, and are generally considered to be safe. However, radiation dose can become a concern in the nuclear and X-ray-based imaging modalities that use ionizing radiation. Ionizing radiation can have a range of acute, latent, and genetic effects with clinical outcomes ranging from mild sickness to sterility to acute illness with early death [1]. Previous reviews have also investigated clinically relevant radiation-induced cancer and skeletal changes that can occur [2, 3].

X-ray micro-computed tomography (μCT) is an imaging modality that is widely used to image both hard and soft tissues for studies in orthopedics, pulmonology, oncology, cardiology, radiology, and infectious disease [4–7]. By directing the X-ray beam at tissue and placing a detector on the opposite side, an image can be created that is directly related to the absorption and scatter within tissue (due to the photoelectric effect and Compton scatter) that leave energy behind in the tissue, creating the dose. Unfortunately, there is no single accepted unit for X-ray dose. Previous studies have used Roentgens (R), Sieverts (Sv), Gray (Gy), radiation absorbed dose (rad), and Roentgen equivalent man (REM) [1, 8]. Irrespective of which unit is used, several fundamental physical concepts contribute to dose. The inverse square law states that dose is inversely proportional to the square of the distance between the source and object. The linear attenuation coefficient is energy dependent, and commonly used sources have a polychromatic X-ray spectrum, so dose is proportional to the sum of all photons absorbed at energies in the spectrum being used. Mathematically, combining these gives the following relationship:

$$ \mathrm{Total}\ \mathrm{dose}\propto \frac{{\displaystyle {\sum}_{\mathrm{Emin}}^{\mathrm{Emax}}}{N}_E\left(L,\mu (E)\right)}{r^2} $$

where

- *E* = X-ray photon energy
- *r* = distance between source and object
- *N*_E_ = # of absorbed photons for a given energy
- *L* = path length that X-rays travel through tissue
- *μ*(*E*) = linear attenuation coefficient

The inclusion of *L* and *μ* in this relationship indicates that there is some dependence on the object itself. The linear attenuation *μ*(E) is tissue dependent. Materials with a higher electron density will attenuate more [9]. Due to the *L* term, smaller objects will tend to have a higher mean dose at the isocenter because of the shorter path length between the center and outside air, but still have a lower total absorbed dose because of the shorter total path length [10].

Beyond this conceptual relationship, there are many sources of noise in X-ray systems that must be considered in practice to achieve the desired image quality for a given application. The simplest explanation based on statistics (SNR ∝ √N, where SNR is the signal to noise ratio and *N* is the number of photons) dictates that dose must increase as the total number of pixels increases to maintain a given per-pixel SNR. More complex models that incorporate a full set of system parameters have shown this to be true [11]. Therefore, guidelines exist to navigate the different tradeoffs (e.g., [12]). In practice, one of the best ways to design imaging protocols is to use phantoms to find the right balance between an acceptable SNR, resolution and dose with the overarching principal of using a dose as low as reasonably achievable (ALARA) [5, 13–16].

Understanding the biological response to radiation is obviously crucial to design longitudinal studies that require ionizing radiation. Fortunately, a number of excellent reviews have been written on the molecular mechanisms governing the response. X-rays can interact with any molecule and, therefore, can cause both short-term and long-term harm. X-ray interactions with water can create reactive oxygen species (ROS) and free radicals which have a number of potential effects on cellular behavior [17]. X-rays can also interact with genetic material and can directly induce damage foci that can be detected with markers such as ATM (ataxia telangiectasia mutated), 53BP1 (p53 binding proten 1), and γH2AX (a phosphorylated version of histone H2AX) [18]. ATM and γH2AX are part of complex signaling cascades leading to DNA repair by homologous recombination (HR) or non-homologous end-joining (NHEJ) processes [19, 20], and these signaling cascades can also be impacted by micro-RNAs [21]. Even low-dose radiation hypersensitivity (defined as doses below 0.3 Gy) can affect both DNA repair and the cell cycle [22]. Therefore, it is not surprising that μCT scans with doses as low as 150 mGy in mice have been reported to induce double-strand DNA breaks [23]. Fortunately, some of these double-strand breaks will start to repair in the initial minutes post radiation and may be largely repaired within the first day, although immune competent C57Bl/6 mice repair more damage than severe combined immune deficient (SCID) mice [23, 24]. Disruptions to the HR and NHEJ signaling cascades or processes could alter these rates.

For preclinical X-ray imaging, it is generally believed that the LD50/30 (the dose lethal to 50 % of the subjects within 30 days) for rodents is in the range of 5–7.6 Gy [11], although this may be reduced to 3 Gy in SCID strains [25]. Radiosensitivity is also dependent on animal strain [26]. Given the complex interplays between physics and biology outlined above, even formal animal welfare guidelines do not provide actual numbers and simply state that doses should be minimized [27]. It is important to look at dose on a tissue by tissue basis and, in some cases, on a study by study basis to understand the potential acute and longer-term impacts.

### Dose Effects: Lung Applications

Lung tissue may not be particularly susceptible to the high doses common in conventional fractionated radiotherapy and frequent μCT scanning. Immunocompetent C57/Bl6 mice scanned three times per week for a 6-week period, with a total cumulative dose of 5.04 Gy, do not have a difference in lung parenchyma volume or Hounsfield unit (HU) value [28]. Similarly, doses on the order of 5–12 Gy delivered to the lung over 5 weeks, or 12 Gy over 12 weeks, do not result in a change in aerated lung volume, lung tissue volume, total lung volume, or gross histopathological change in immunocompetent C57Bl/6 mice [29]. However, very high doses for lung imaging could still be problematic. One week after delivery of a 90 Gy dose to the lungs of immunocompetent C57Bl/6 mice for sterotactic body radiotherapy there is no noticeable change in gross morphology or lung structure on a μCT image, although there is histopathological damage that will eventually lead to fibrosis and altered lung function as early as 2–3 weeks later [30]. The 30 Gy dose threshold to induce this fibrosis in immunocompetent C57Bl/6 mice is slightly lower for than the 50 Gy needed to induce fibrosis for immunocompetent C3H mice [31]. A single 20 Gy dose to the lungs of immunocompetent C57Bl/6 mice does not cause a change in lung volume manually measured on a μCT image after 4 days, but the HU value shifts and air spaces are enlarged [32]. However, the simple image analysis methods typically used for lung studies may not be adequate to detect all of the changes that are visible in μCT images. A variogram-based image analysis approach may be needed to capture the effects of 6–15 Gy doses [33]. Most importantly, since lung imaging is often used for cancer studies, an investigator must keep in mind that specific genetic mutations can have differential effects. As one example, the presence or absence of a tumor suppressor gene in mice impacts susceptibility to the difference between a single 11.6 Gy dose and a fractionated 14.6 Gy dose delivered over 2 days [34].

### Dose Effects: Bone and Orthopedic Applications

Bone tissue has a higher linear attenuation coefficient than soft tissues and experiences local doses approximately 3–5 times higher than lung tissue [29, 35, 36]. Therefore, many studies have investigated the effects of ionizing radiation on bone in a variety of environments. Within the context of a radiation accident, a single 8 Gy dose of ^60^Co irradiation to immunocompetent B6D2F1/J mice induces weight loss, decreases trabecular bone volume fraction and reduces the bone formation rate as long as 120 days post-exposure [37]. In the context of spaceflight, a 1–2 Gy dose of common particles (gamma rays, protons, ^12^C^6+^, ^56^Fe^26+^) to immunocompetent C57Bl/6 J mice decreases the trabecular bone volume fraction and volumetric bone mineral density (vBMD) over 110 days even though animal weight does not change [38, 39]. Two Gy of gamma irradiation in C3H/HeN mice decreases the trabecular bone volume fraction and BMD as early as 12 weeks post-irradiation [40]. In contrast, the delivery of 4.4 cGy to the appendicular skeleton (a subset of the 60 Gy delivered to the head) of immunocompetent C57Bl/6 mice results in a remarkable increase in trabecular BV/TV 11.5 months after exposure [41].

For X-ray radiation there are still potential issues for normal μCT imaging protocols. Increased scan doses may be required if high resolutions are needed for accurate bone morphology measurements [42]. These doses can alter the experimental outcomes. A 712 mGy dose delivered almost weekly (1, 2, 3, and 5 weeks) to immunocompetent C3H/HeJ, C57Bl/6J, or BALB/cByJ mice both with and without ovariectomy (OVX) results in a trabecular BV/TV decrease, but immunocompetent Wistar rats imaged every other week do not show the same effect [43]. Similarly, imaging immunocompetent Wistar rats weekly with a dose between 441 and 939 mGy over a 7-week period does not change the trabecular BV/TV [44]. For mice, the time interval might be more critical. One study reports a 30 % loss in trabecular BV/TV of immunocompetent C57Bl/6 mice with three 776 mGy scans separated by 2-week intervals, but not when the dose per scan was reduced to 434 mGy [45].

The radiation-induced changes detected in these μCT imaging studies are probably reflective of phenomena that are more systemic and cellular in nature. Single 15–20 Gy doses of X-rays delivered to the abdomen of immunocompetent C57Bl/6 mice results in a BMD decrease in the femur and tibia as early as 7 days post-radiation [46]. The same study also reports a weight decrease 5–7 days after irradiation, similar to other data indicating a significant weight decrease in immunocompetent C57Bl/6 and BALB/c SCID mice 3 days after a 3.36 Gy dose [23]. Both a 0.5 and 2 Gy whole body dose of X-ray radiation to immunocompetent C57Bl/6JJcl mice results in fewer bone marrow cells as early as 1 day post-exposure [47], and a 2 Gy whole body dose of X-rays to immunocompetent C57Bl/6 mice increases osteoclast number as early as 3 day post-exposure [48]. Osteophytes can occur with a whole body dose as low as 3 Gy in immunocompetent Fisher F344 X Brown Norway rats, and cartilage degradation worsens as dose increases [49]. Localized dose delivery can also be problematic. Both 5 and 20 Gy of X-rays delivered to one limb of immunocompetent Balb/c mice induces alopecia and erythema, and results in a dose dependent decrease in trabecular BV/TV 6 weeks post-irradiation [50]. Trabecular bone volume losses in both irradiated and the non-radiated contralateral tibiae 10 days after a 2 Gy dose of X-ray radiation localized to the limb of immunocompetent C57Bl/6 mice is also associated with a body weight decrease attributed to fat loss, increased marrow adiposity, increased number of osteoclasts, and reduced bone formation rate [51].

Commercially available pharmaceuticals may be able to help prevent the effects of radiation-induced bone loss from X-rays. Treatment with the anti-catabolic bisphosphonate risedronate results in an increased trabecular BV/TV 3 weeks after a 2 Gy dose of X-rays to immunocompetent C57Bl/6 mice, even though the radiation alone reduces BV/TV and vBMD as early as 1 week and decreases the bone formation rate starting at 2 weeks [52]. The anabolic agent parathyroid hormone (PTH) 1–34 increases vBMD and BV/TV starting 4 days after treatment in immunocompetent Sprague Dawley rats, even though the 0.48 Gy dose delivered during daily imaging decreases vBMD and BV/TV without PTH [53].

### Dose Effects: Oncology Applications

Damage from accumulated radiation is a particular concern in cancer research, especially since longitudinal monitoring is often essential for tracking tumor progression [34, 54–56]. Given that radiotherapy is a common approach for treating cancer, it is not surprising that high local doses of radiation can reduce tumor volume [57–59]. This must be done with caution. Doses of 2–4 Gy of gamma radiation to hepatocellular carcinoma cells *in vitro* can increase the stemness of cancer cells after 7–14 days [60]. A single 30 Gy X-ray dose delivered directly to a tumor causes a significant upregulation of genes associated with angiogenesis and downregulation of genes for eukaryotic initiation factors [61]. This may be related to changes in vascularity. A single 20 Gy dose of X-ray radiation to sarcomas in transgenic mice increases the fractional blood volume and dextran accumulation within a sarcoma after just 4 days [57]. This is about the same timepoint at which vasculature within a tumor begins to reform [61]. The MET oncogene is upregulated *in vitro* by a 10 Gy dose of X-rays with the resulting cells being able to migrate easier [62]. A 5 Gy dose to subcutaneous tumors implanted in athymic nude mice may be sufficient to induce DNA damage in a tumor and induce a growth delay [59]. However, a 3 Gy dose of X-rays delivered locally to xenografts implanted in nonobese diabetic–severe combined immunodeficient (NOD/SCID) mice results in a significant weight loss by the 5th day post-irradiation [58], and 3–10 Gy of X-ray radiation to cancer cells *in vitro* may induce tumor cells to repopulate [63].

Lower radiation doses that are more common in imaging procedures can also be problematic. X-ray doses as low as 0.25–2 Gy induce intestinal and colon tumors in genetically prone immunocompetent mice in an age and strain-dependent fashion [64]. Doses as low as 0.3–0.5 Gy to human microvascular endothelial cells (HMVEC) can induce molecular markers of vascular formation, especially in a simulated hypoxic environment, and this can lead to increased angiogenesis in athymic swiss *Nu/Nu* mice and an increased tumor burden 14 days later in an orthotopic NOD/SCID murine model [65]. Performing a ~ 1 Gy scan in a metastatic tumor model using athymic nude mice once weekly over a 5-week period increased leg metastasis and the histological tumor area nearly two-fold [56]. Conversely, although high-resolution scans may kill tumor cells, when the imaging protocols are designed to manage dose there may not be a detrimental effect on bone tumor growth with weekly scans of athymic nude mice over a 4-week study [54]. Doses of 0.07–0.30 Gy every 4 days during a routine imaging procedure also do not change the volume of subcutaneous tumors implanted in immunocompetent C57Bl/6 mice after 16 days [55].

Radiation effects on the immune system may also need to be considered in oncology studies utilizing μCT imaging. Many preclinical oncology studies use SCID mice which have increased radiosensitivity and a defective DNA repair mechanism [26, 66]. Tumors derived from SCID mice are more susceptible to high doses of radiation than tumors derived from C3H mice [25], which may be related to endothelial biology [67]. Conversely, translational oncology studies that use immunocompetent rodent strains are becoming increasingly important as immunotherapies that impact NK, T, and B cells of the immune system gain traction [68]. Individual cytokines like IL-1β that are relevant in immune system signaling may be upregulated by high doses of X-ray radiation [69]. At doses more relevant to *in vivo* μCT imaging, 0.1 Gy of 80 kVp X-ray radiation significantly alters gene regulation in these immunological pathways [70]. Immunologically relevant cytokines and the cytolytic activity of NK cells and macrophages is also increased in response to a single dose of 0.1 or 0.2 Gy of X-rays, which can potentially act as a radioprotectant by reducing subsequent tumor burden [71].

### Dosimetry Methods

Given these complicated effects and interactions, it is clearly important to have a precise understanding of radiation dose within a scan. In clinical environments, several standards have been implemented to ensure reproducibility and patient safety [72–75]. Unfortunately, there is no similar standard for preclinical research. A number of different techniques have been applied in the literature. The simplest and most widely accepted approach is to use an ionization chamber [10, 13, 28, 29, 33, 39, 43–45, 55, 58, 76–79]. This data can then be used to calculate the CT dose index (CTDI_100_) as is commonly done in clinical environments. Gafchromic films can be used to develop a depth profile if more detailed information is needed [14, 23, 61, 80]. Others have implanted thermoluminescence dosimeters (TLDs) or nanoDots near specific organs within an animal to measure the doses at specific locations [14, 29, 42, 76, 77, 81–87]. The most complete map of dose in every tissue requires Monte-Carlo-based simulations, although the implementation complexity and variability in the results makes these techniques challenging to reproduce [10, 29, 35, 36, 76].

In light of the complex relationships between dose and experimental outcomes, to help guide proper study design and application of μCT imaging methodologies, the purpose of this work is to characterize dose on the Quantum GX and provide a framework for protocol selection. Ionization chambers are used to measure the dose at the center of x-ray beam and CTDI_100_ on the rotation axis. The results can be appropriately used in a translational context. Doses from other μCT imaging systems will also be described to provide some context for where this newer system compares with other scanners used in the scientific literature.

## Experimental Design and Methods

### Scanner Configuration

The Quantum GX uses a complementary metal-oxide semiconductor (CMOS)-based flat-panel detector. Various imaging modes within the scanner enable this to be used with bin modes of 1, 2, or 4. The flat panel detector is mounting on an adjustable magnification mechanism which, in turn, is mounted on a rotating gantry. This adjustable magnification mechanism allows the source to object distance to be modified, enabling a broader range of applications to suit both high-resolution and low-dose applications. The source to detector distance is fixed. The actual distances and detector specifications are reported in Table 1.

**Table 1:** Specifications of the Quantum GX imaging system that are used in CTDI_100_ calculations

Specification	Value
Detector pixel pitch	49.5 μm
Number of rows on detector (*z*-axis)	2352
Number of columns on detector (*x*, *y*-axes)	2944
Source to detector distance (SDD)	203 mm
Source to object distance (SOD), high magnification (36mm acquisition FOV)	55 mm
SOD, low magnification (72 mm acquisition FOV)	108 mm

The microfocus X-ray source in this scanner is limited to a maximum voltage of 90 kVp and a maximum power of 8 W. The X-ray source uses a Tungsten anode. A fixed filter of 0.5 mm Al and 0.06 mm Cu is placed in front of the exit port to remove low energy X-rays that contribute to dose but do not improve image quality.

### Half Value Layer Measurements

The half value layer (HVL), defined as the thickness of aluminum that reduces the radiation energy by 50 %, was measured to get a sense of the X-ray beam quality. A meter was placed at isocenter of the X-ray beam on a sample bed normally used for rat imaging (Piranha 657, RTI Electronics AB). Data were measured for voltage settings of 50, 60, 70, 80, and 90 kVp. Both the measured voltage and half value layer were reported.

### Dosimetry Measurements

**Fig. 1:**
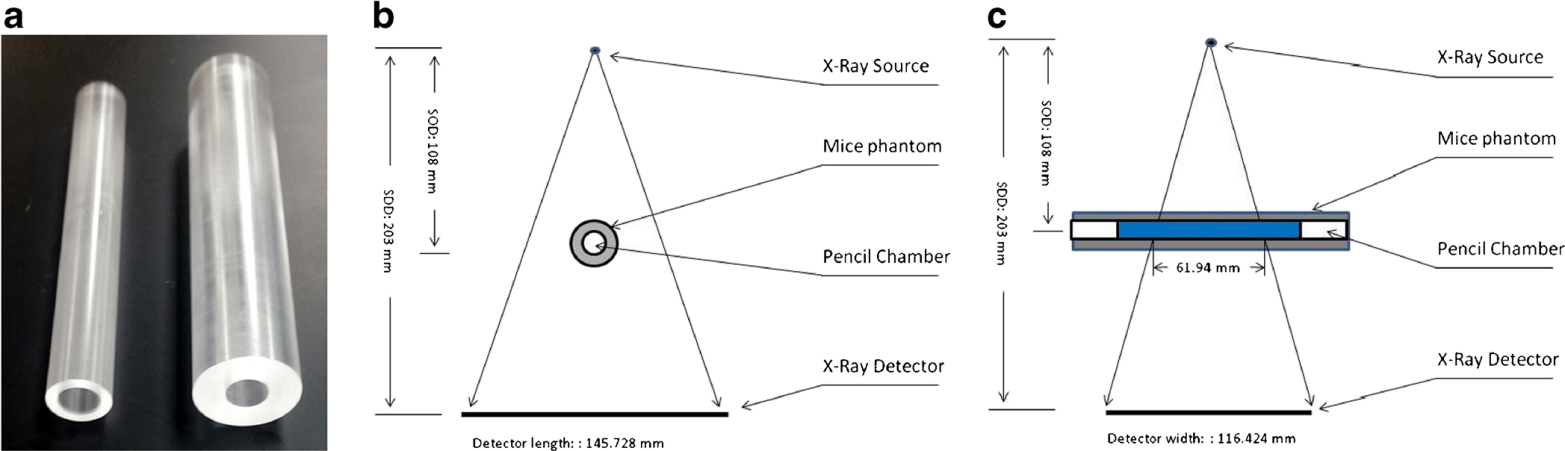
**a** PMMA phantoms placed around the ionization chamber for measuring doses relative to a mouse and rat. **b** Front view of dose measurement set-up in low magnification mode showing the system geometry listed in Table 1. **c** Side view of dose measurement set-up in normal mode. The total x-ray beam width at rotation axis is defined as *N* × *T* × SOD/SDD, where the parameters are defined in Table 1. For the low magnification mode shown here this equates to 0.0495 × 2352 × 108/203 = 61.94 mm. For the high magnification mode (not shown) this equates to 0.0495 × 2352 × 55/203 = 31.543 mm.

Two cylindrical phantoms were made from polymethyl methacrylate (PMMA) with outer diameters of 20 and 32 mm (Fig. 1a) to mimic attenuation from a mouse and rat, respectively, similar to previously reported studies [76]. The inner diameter of 12.54 mm was made to fit the 10-cm-long pencil ionization chamber used for dosimetry measurements. The phantom length was 15 cm to be consistent with clinical dosimetry practices [75].

To make the measurements, the ionization chamber was either placed directly on the sample bed or slid into the phantom before being centered on the sample bed. The sample bed was then moved so that the center of the ionization chamber was aligned with the z-center of the X-ray beam (Fig. 1b). A dose measurement was then made for a fixed tube power of 7.9 W at voltage of 50, 70, or 90 kVp. Measurements were made in triplicate and the mean value was used for reporting doses. A schematic set-up for the dose measurement in low magnification scanning mode is shown in Fig. 1c.

A Thimble chamber (Model 10 × 5–0.6CT, Radcal Inc., Monrovia, CA, USA) was used to measure the dose at the center of the x-ray beam on the rotation axis. The chamber has an active length of 21 mm and an active volume of 0.6 cm^3^. A pencil ionization chamber (Model 10X5-3CT, Radcal Inc., Monrovia, CA, USA) was used to measure CTDI_100_ on the rotation axis. Both chambers have flat energy response with about 4 % variation over half value layers (HVL) between 1.6 and 10 mm of aluminum (Radcal Calibration Laboratory Personal Communication) Each chamber was connected to a dose monitor (Model 9015, Radcal Inc., Monrovia, CA, USA) for reading out. The calibration of the dosimeter system was traceable to the National Institute of Standards and Technology (NIST).

### CTDI_100_ Calculation

Once the raw measurements were completed, CTDI_100_ was calculated as defined in units of mGy using the equation

$$ {\mathrm{CTDI}}_{100}=\frac{1}{N\ast T}{\displaystyle {\int}_{-50\kern0.5em  mm}^{50\kern0.5em  mm}D(z)dz} $$

where *N* is defined as the number of slices, *T* is defined as the slice thickness at ISO, and *D*(*z*) is the dose profile along the *z*-axis (Fig. 1c). The product of N*T is effectively the beam width in the *z*-direction at the isocenter. Because the ionization chamber measures an average exposure (in units of mR), the f-factor was included to convert the exposure to the actual absorbed dose in air (in units of rad) and converted into mGy. CTDI_100_ measurements were then normalized to mA × s based on the acquisition times. The f-factor of 0.87 rad/R was used [74]. Even though this value is noted for 120 kVp, the same value will be used for the softer beams here because the responses are almost constant with respect to energy [88].

### Image Acquisition and Analysis for Application Examples

Images of mice and phantoms were obtained to study the relationship between dose and image quality. For animal images, 7-month old immunocompetent female BALB/c mice were used. One mouse was scanned at peak X-ray tube voltages of 50, 70, and 90 kVp for the shortest scan time using the low magnification. A second mouse was scanned at 90 kVp using all available scan times applicable for *in vivo* imaging, and then a single slice was reconstructed at the highest allowable resolution. A ring reduction correction was applied to the sinograms, and the resulting corrected sinograms were input into a GPU-based filtered-backprojection reconstruction algorithm using a Ram-Lak filter. Images were then imported into Analyze 12.0 (AnalyzeDirect, Overland Park, KS) and calibrated to be on the Hounsfield unit (HU) scale. A segmentation map was created using semi-automatic segmentation tools to select voxels of bone, adipose tissue, lung parenchyma, and general soft tissue (areas of the GI tract containing food were excluded). This segmentation map was then applied to each image to calculate the mean HU value for each tissue type. The contrast to noise ratio (CNR) was also calculated for each tissue type. Contrast was defined as the difference between the mean value of tissue and the mean grayscale value of the animal bed. Noise was defined as the standard deviation of air. All experiments were done under the approval of an institutional animal care and use committee.

Two separate phantoms were also scanned at a peak X-ray tube voltage of 90 kVp to explore the relationships between dose and image quality. The first phantom was a 32 mm diameter low contrast phantom that contains 1 and 2.5 mm diameter rods embedded in resin at an approximate contrast 4 and 8 % HU (QRM-MicroCT-LC, QRM GmBH, Möhrendorf, Germany). The second phantom was a 32 mm diameter hydroxyapatite (HA) phantom typically used for bone mineral density calibrations that contains materials equivalent to 0, 50, 200, 800, and 1200 mg/cm^3^ of HA embedded in resin (QRM-MicroCT-HA, QRM GmBH, Möhrendorf, Germany). The scan times were selected to be both 18 s and 2 min, two protocols in the Quantum GX which use a 2 × 2 detector binning mode. A ring reduction correction was applied to the sinogram, and the resulting corrected sinogram was input into a GPU-based filtered-backprojection reconstruction algorithm using a Ram-Lak filter to create the 3D images. Images were then imported into Analyze 12.0, calibrated to be on the HU scale, and visualized.

## Results

### Quantum GX Dose Measurements

Half value layer measurements ranged from 1.56 mm Al for 50 kVp up to 2.6 mm Al for 90 kVp (Table 2). The HVL measurements increase as a function of peak X-ray source voltage. As confirmed by the dosimeter vendor Radcal, the energy dependency of the ionization chambers is flat in the measured HVL range with a maximum variation of 4 %.

**Table 2:** X-ray source settings and half value layer measurements

Voltage setting (kVp)	Current setting (uA)	Tube power (W)	Measured voltage (kVp)	HVL (mm Al)
50	158	7.90	49.57	1.56
60	133	7.98	60.24	1.71
70	114	7.98	70.18	1.96
80	100	8.00	79.93	2.26
90	88	7.92	89.35	2.60

The measured CTDI_100_per mAs at the center of x-ray beam on the rotation axis are shown in Table 3. For both the low- and high-magnification modes, the difference between the measured CTDI_100_ in air, in the mouse phantom, and in the rat phantom is less than 13 % for a given kVp setting. Interestingly, for 90 kVp with the low magnification, the dose rate increased slightly in comparison to air when the mouse phantom was used but decreases again when the rat phantom was used. Dose rates are also a function of the peak X-ray voltage. The CTDI_100_ for 70 kVp are 36–40 % lower than at 90 kVp. The CTDI_100_ for 50 kVp are 70–73 % lower than at 90 kVp. The middle point dose in air was measured at 7.0 mGy/mA*s for a 70 kVp beam and 11.8 mGy/mA*s for a 90 kVp beam.

**Table 3:** Dose rate measurements on the Quantum GX μCT imaging system

Magnification	Environment	CTDI_100_ measurements (mGy/mA*s)		
		50 kVp, 158 uA (7.90 W)	70 kVp, 113 uA (7.91 W)	90 kVp, 88 uA (7.92 W)
Low (72 mm FOV)	Air	3.49	7.72	12.07
	20 mm PMMA (mouse)	3.38	7.33	12.21
	32 mm PMMA (rat)	3.12	6.89	11.48
High (36 mm FOV)	Air	14.31	30.07	48.65
	20 mm PMMA (mouse)	13.74	29.53	47.57
	32 mm PMMA (rat)	12.45	27.68	45.54

For the shortest 8-s scan time on the Quantum GX, at 90 kVp the dose floor (defined as the lowest possible dose for a particular configuration) is 39.8 and 9.9 mGy, respectively, for the high and low magnifications in air. The dose floor reduced slightly to 9.4 mGy when the 32 mm PMMA phantom was used. Reducing the tube voltage to 50 kVp changes the dose floor to 21.0 and 5.1 mGy, respectively, for the high and low magnifications in air.

### Application Examples

**Fig. 2:**
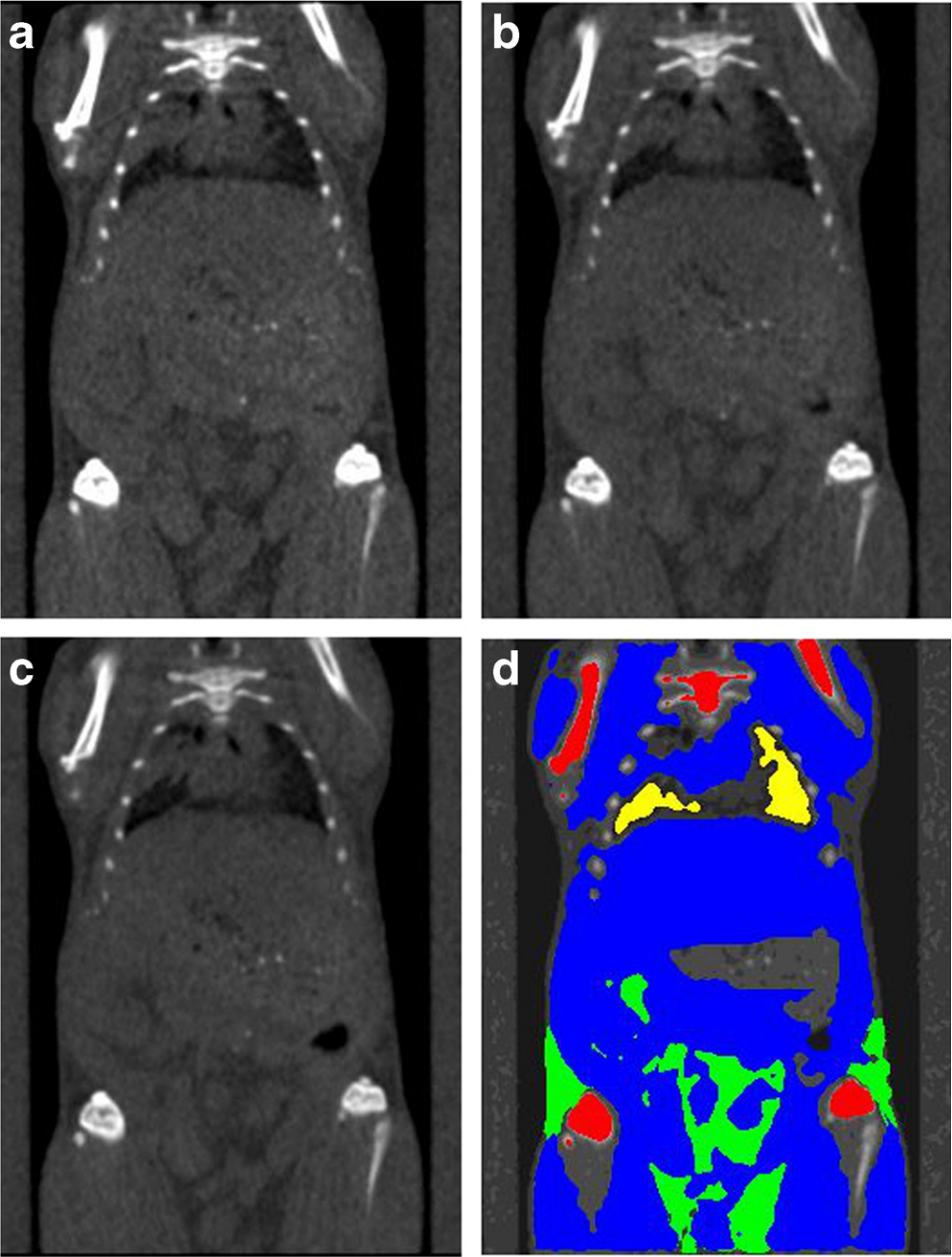
Example coronal slices showing the shortest 8-s scan using the low magnification for peak X-ray source voltages and doses of **a** 50 kVp and 5.0 mGy with a noise level of 38 HU, **b** 70 kVp, and 7.8 mGy with a noise level of 34 HU, and **c** 90 kVp, and 10.0 mGy with a noise level of 32 HU. Doses were calculated using the CTDI_100_ measurements for the mouse equivalent phantom. **d** The bone *(red*), lung parenchyma (*yellow*), adipose tissue (*green*) and soft tissue (*blue*) were segmented, and the mean grayscale values for each mask were calculated (Table 4). The GI tract was specifically excluded because chow could potentially bias the soft tissue grayscale value. All images are displayed with a level of 750 HU and a window of 2500 HU, and filtered with a 3 × 3 ×3 low pass filter to reduce noise.

**Fig. 3:**
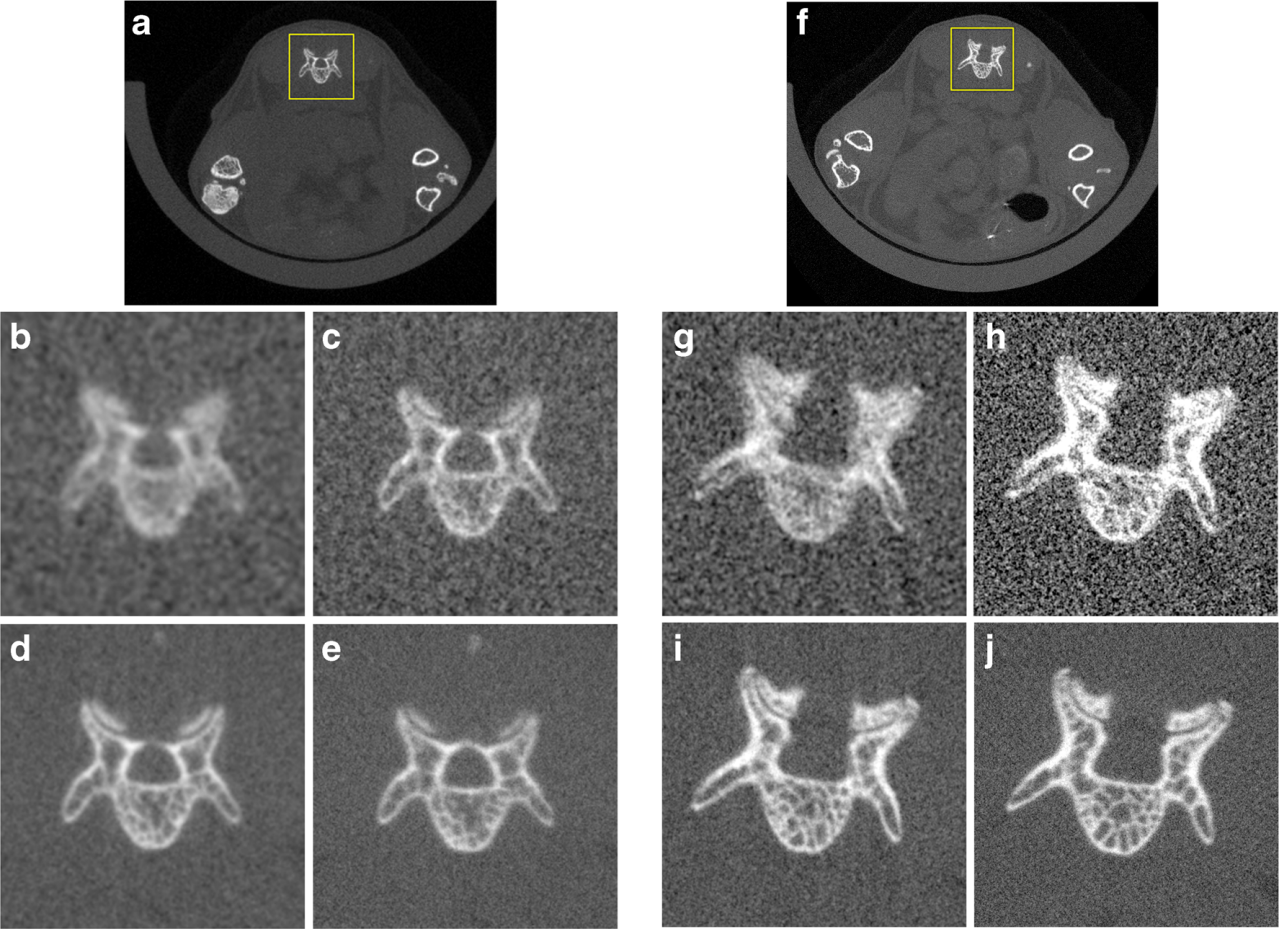
Example images of a transaxial rostral slice through L6 showing subvolume reconstructions at the highest resolution possible. **a** The low magnification was then cropped (*yellow box*) for display purposes to show the **b** 8-s scan, 36 μm isotropic voxel size, 10 mGy, **c** 18-s scan, 18 μm isotropic voxel size, 22.4 mGy, **d** 2-min scan, 18 μm isotropic voxel size, 130.4 mGy, or **e** 4-min scan, 9 μm isotropic voxel size, 269.1 mGy. **f** The high magnification was then cropped (*box*) for display purposes to show the **g** 8-s scan, 18 μm isotropic voxel size, 38.9 mGy, **h** 18 s scan, 9 μm isotropic voxel size, 87.1 mGy, **i** 2-min scan, 9 μm isotropic voxel size, 508.2 mGy, or **j** 4-min scan, 4.5 μm isotropic voxel size, 1048.2 mGy. All images are displayed with a level of 1000 HU and a window of 4000 HU with no filtering applied.

A series of example images were acquired to provide context for interpreting how dose may impact applications. For screening applications that typically use the low-dose protocols, a single mouse was scanned using a range of common X-ray source voltages. A segmentation map of bone, adipose tissue, soft tissue, and lung parenchyma were created and applied to each of the images (Fig. 2). There is an increase in contrast of bone as the voltage decreases (Table 4). There was no measureable change in HU value as a function of voltage for adipose tissue, soft tissue, or lung parenchyma. The noise level and CNR did change slightly for soft tissue and lung parenchyma as a function of voltage. There were changes for bone applications that typically require high resolution and necessitate higher doses, a single mouse was scanned using a range of scan times and the best resolution image possible for each scan time was reconstructed (Fig. 3). There is a significant increase in anatomical detail as the scan time increases and as the physical magnification increases.

**Table 4:** Grayscale values in HU (mean ± sd) and CNR for the images shown in Fig. 2

Tissue type	50 kVp		70 kVp		90 kVp	
	Grayscale /	CNR	Grayscale	CNR	Grayscale	CNR
Adipose	–23 ± 96	–1.7	–40 ± 74	–2.4	–42 ± 71	–2.4
Soft tissue	159 ± 84	3.0	116 ± 68	2.2	92 ± 62	1.7
Lung parenchyma	–275 ± 80	–8.3	–298 ± 79	–9.9	–308 ± 77	–10.6
**Bone**	2118 ± 650	54.3	1623 ± 502	46.4	1339 ± 438	40.4

**Fig. 4:**
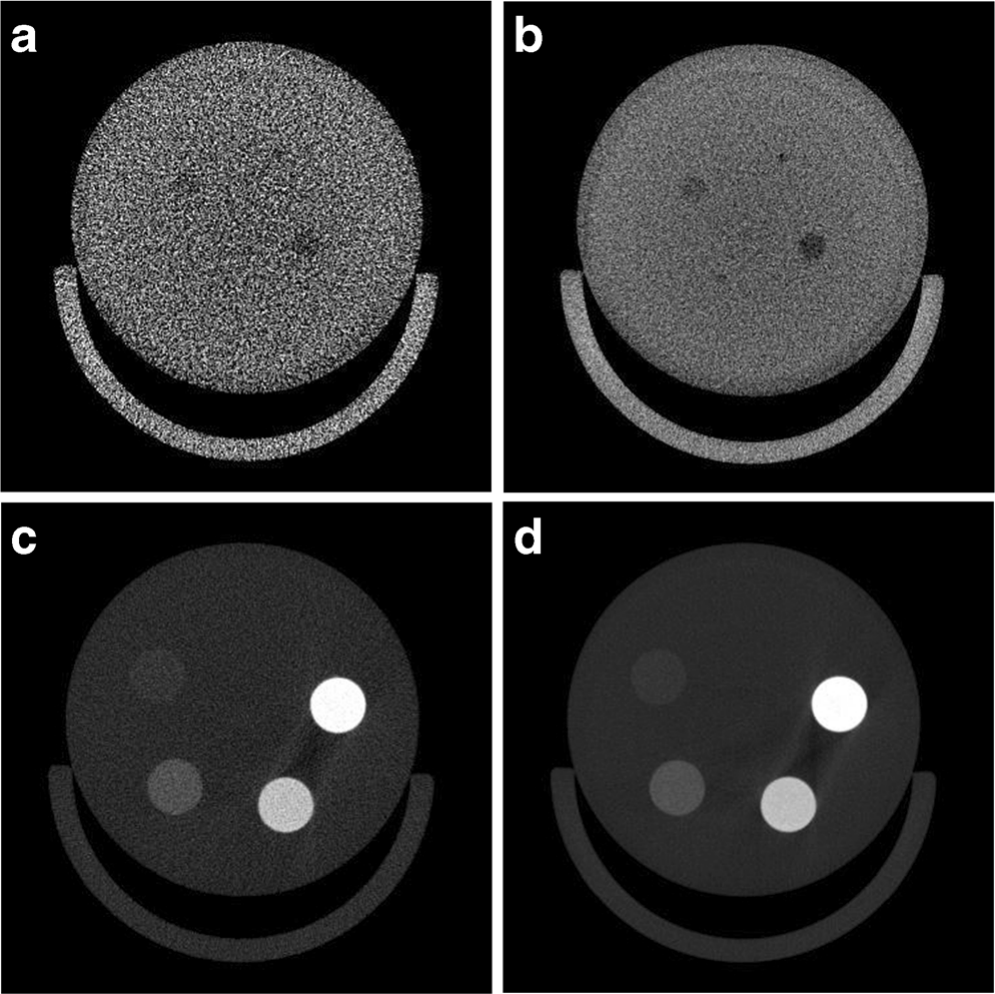
Comparisons of **a**, **b** a low contrast phantom displayed with level of 0 HU and a window of 400 HU and **c**, **d** a HA phantom displayed with a level of 1000 HU and a window of 3000 HU. The images were acquired with either **a**, **c** an 18 s, 22.4 mGy scan or **b**, **d** a 2 min, 130.4 mGy scan using 2 × 2 binning on the detector, a 90 kVp X-ray source voltage and the low physical magnification. Increasing the dose by increasing the scan time enables visualization of all four inclusions in the low contrast phantom. All four inclusions were visible in the HA phantom at either scan time.

To provide an example of how dose relates to image quality for a given detector bin mode, voltage and physical magnification, two separate phantoms were scanned. In a low-contrast phantom, a 1 mm insert with an approximate HU contrast 4 % was not visible with a 18-s scan but was visible with a 2-min scan (Fig. 4a, b). All other rods in the phantom were much easier to visualize when the scan time increased. For a HA phantom that contains a range of mineral density equivalents, all four inclusions containing HA could be easily visualized at both scan times (Fig. 4c, d).

### Dose from Other Scanners

In order to have context for the dose data for the Quantum GX imaging system, a literature review was performed to get a sense of the doses reported for other similar instrumentation (Table 5). Dosimetry methodologies vary and can have considerable impact on how the dose is reported, so the methods used are noted along with the data. Many instruments have a wide range of doses reported because there is considerable flexibility in how the system is used. A dose floor below 10 mGy is reported for two instruments (BioScan nanoSPECT/CT and Aloka LaTheta LCT-200), below 100 mGy for five instruments (GammaMedica X-O, GE CT120, GE Locus Ultra, Imtek MicroCAT II, Siemens Inveon), and above 100 mGy for three instruments (ScanCo vivaCT40, Skyscan 1076, Skyscan 1178) (Table 4). Comparing performance between systems is difficult because of differences in system design and the lack of a standardized approach. Therefore, the primary application for each publication was collated. While there is a considerable range of doses, orthopedics applications generally have higher doses while applications in general radiology for soft tissue screening typically have lower doses. It should be noted that other systems are available and that various manufacturers may have different data than what is presented here. The data reported in Table 5 is based solely on peer-reviewed publications.

**Table 5:** Dose measurements for μCT imaging systems reported in the peer-reviewed literature

CT instrument	Dosimetry methods	Imaging application Area	Doses reported	Ref.
BioScan nanoSPECT/CT	EBT2 gafchromic films	Multi-modality, oncology	8.3–3361 mGy	[23]
CT Imaging TomoScope dual source scanner	CTDI_100_ (ionization chamber) LiF TLDs Monte Carlo simulations	General	153–200 mGy	[76]
Gamma Medica X-O	(Not reported)	Pulmonology	63.6 mGy	[91]
Gamma Medica X-SPECT/CT	(not reported)	Multi-modality, oncology	~1000 mGy	[56]
Gamma Medica X-SPECT/CT	LiF TLDs	Oncology	14–134 mGy	[85]
GE CT120	CTDI_100_ (MOSFET)	General	20.15–56.79 mGy	[101]
GE CT120	Monte Carlo simulations	General	Whole body, mouse: 67–189 mGy Whole body, rat: 42–119 mGy	[36]
GE eXplore Locus	LiF TLDs	General	126–229 mGy	[87]
GE Locus Ultra	Ionization chamber	General	64–270 mGy	[13]
GE Locus Ultra	Ionization chamber	Oncology	69–296 mGy	[55]
GE RS—90	Ionization chamber	Pulmonology	Ungated: 120 mGy Gated: 150 mGy	[78]
Imtek MicroCAT	TLDs	Oncology	Organs: 210–760 mGy	[86]
Imtek (Siemens) MicroCAT II	LiF TLDs ionization chamber	General	Air: 78–97 mGy Organ: 65–86 mGy	[81]
Imtek (Siemens) MicroCAT II	NanoDot	General	11–104.5 mGy	[83]
Imtek (Siemens) MicroCAT II	TLDs	General	Air: 55.6–61.5 mGy	[84]
LaTheta LCT—200	TLDs	Contrast agents	5–40 mGy	[15]
Rigaku RmCT2 (PerkinElmer Quantum FX)	FGDs	General	16 mGy	[79]
ScanCo vivaCT40	Ionization chamber	Whole body scanning (mice)	Polymer: 712.4 mGy Air: 845.9 mGy	[43]
ScanCo vivaCT 40	CTDI (ionization chamber)	Orthopedics	Air: 939 mGy Polymer: 441 mGy	[44]
Siemens Inveon	TLDs	Orthopedics	40–1257 mGy	[42]
Siemens Inveon	NanoDot	General	Organs: 9.4–163.1 mGy	[83]
Skyscan 1076	(Not reported)	Orthopedics	400 mGy	[102]
Skyscan 1076	Ionization chamber TLDs Monte Carlo	Pulmonology	Ionization chamber: 1640 mGy TLDs, skin: 1104 mGy TLDs, lung: 813 mGy Monte Carlo, organs: 900 mGy–4.5 Gy	[29]
SkyScan 1076	Ionization chamber	Orthopedics	166, 434 or 776 mGy	[45]
Skyscan 1178	CTDI_100_ (ionization chamber) LiF TLDs	General	Organs: 295–507 mGy	[77]
X-Strahl SARRP	(Not reported)	Radiotherapy planning	16, 32 or 64 mGy	[103]
Custom dual energy system	Not reported-data unpublished	Oncology	260 mGy	[57]

## Discussion

The lowest dose preclinical systems use doses on the same order of magnitude as the 1–10 mSv values typically reported in clinical CT [89]. These doses are considered to be manageable in longitudinal studies. For example, scanning mice three times weekly for a 4-week period with a 16 mGy protocol does not impact body weight, organ weights, or hematological readouts either acutely or when followed by a 4-week latency period [79]. The dosimetry data presented in this study indicate that the Quantum GX has a dose floor that is also within the range of clinical CT systems. For the typical ‘recommended’ voltage of 90 kVp, although there is a wide range of possible doses depending on the imaging mode chosen, the lowest dose is 9.9 mGy. This is similar to two other instruments showing doses below 10 mGy and, in itself, is a conservative estimate because it is based solely on the air kerma rate. The PMMA phantom results indicate that the dose may decrease slightly when mice or rats are scanned. The slight increase in CTDI_100_ in the low magnification when switching from air to the mouse PMMA phantom may indicate a slight increase in scatter that is then absorbed when the thicker rat PMMA phantom is used.

HVL data for the Quantum GX is comparable to the 2.5 mm Al HVL of another preclinical μCT system that uses a 80 kV beam [81]. More importantly for this study, this range is consistent with other radiographic imaging equipment, so ionization chambers are generally applicable. Doses for the lower energies (50 and 70 kVp) may be up to 4 % underreported because the typical clinical CT systems have a slightly higher typical HVL [90], but for HVL values above approximately 2.0 (80–90 kVp) the dosimetry data can be considered accurate. This is reinforced by the similarity between the thimble chamber measurements and the ionization chamber measurements. The dose floor of the Quantum GX is also consistent with other preclinical instrumentation. Direct head to head comparisons between the various preclinical instruments are quite difficult because the system designs differ widely. Differences between the instruments in physical geometry, X-ray beam quality, and operating modes (voltages, scan times, etc.) all have a significant effect on dose. Differences in the detective quantum efficiency due to the scintillator, optical coupling, and sensor type (e.g., charge-coupled device (CCD), CMOS, amorphous Si) also significantly impact how the radiation dose affects image quality in a projection image. Reconstruction implementations including the type of algorithm (e.g., filtered backprojection, iterative) and filter kernel also impact the quality of the reconstructed 3D image. However, the comparative data available in the literature still provide a valuable reference point for understanding what is common within preclinical imaging.

Some preclinical applications may be achievable with low-dose protocols, but some applications may require higher-dose imaging protocols. For general whole body screening, the Quantum GX provides excellent image quality with the lowest dose 50, 70, and 90 kVp protocols. The decrease in contrast for bone tissue in a mouse as the X-ray source voltage increases is consistent with reduced attenuation as the HVL increases. The lack of measurable changes in contrast of adipose tissue, soft tissue, and lung parenchyma as a function of voltage suggests that the HVL may need to be reduced further to increase contrast for these tissues. Alternatively, as demonstrated using a low-contrast phantom, switching to a higher-dose acquisition protocol can help improve the contrast to noise ratio for visualizing low-contrast structures. However, the ability to visualize bone and HA at 90 kVp indicates that low-dose acquisitions may be sufficient for some applications. However, if skeletal morphology needs to be measured, using a higher-dose protocol can substantially improve the ability to resolve small structures [42].

There are also applications specific approaches for gating in cardiopulmonary imaging that can affect dose. Prospective gating methods theoretically have equivalent dose to ungated protocols, although the dose for respiratory applications may still increase approximately 25 % in practice [78]. This is still much lower than retrospective methods that require oversampling and increase dose in comparison to prospective methods [91]. However, this is often acceptable because the retrospective methods will work for variable respiration rates, whereas prospective methods assume that the respiration interval is constant during the entire scan. The only way to ensure that dose for ungated and gated acquisitions are identical is to intubate and mechanically ventilate so that the same protocol can be used [92]. Cardiac gating methods face similar challenges, although the dose for cardiac gated acquisitions can also be lowered if image analysis approaches are designed that are robust against the image quality reductions which occur [93].

There are several possible approaches to reducing dose in the future versions of the Quantum GX. First, because this dose is for an operating mode where data is acquired over 360 degrees, optimizations to use a half scan protocol could reduce this even further [94]. Second, the data presented here suggest that the dose could be reduced quite considerably by switching from 90 kVp to lower voltages and still provide excellent image quality for certain applications. Although the expected gain in soft tissue contrast was not obvious for the shortest 8-s scan time, 50 kVp has been used using the earlier generation Quantum FX for adipose imaging [95], so the lower voltages may still be useful for soft tissue quantification. Further optimizations of the X-ray filters that either reduce the HVL and increase soft tissue contrast, or increase the HVL and further reduce dose, are also plausible. There are also several software improvements that can potentially reduce dose. Clinical CT systems now use fairly sophisticated image reconstruction algorithms, but most μCT systems still use variations of the filtered-backprojection methods initially reported several decades ago [96]. Recent research indicates that more advanced reconstruction methods can be employed which allow the number of projections to be reduced, thereby reducing the required X-ray dose [97, 98].

In the clinical CT environment, the Image Wisely^®^ and Image Gently^®^ initiatives help ensure that the ALARA principle is followed [99, 100]. However, there is no similar initiative or any widely accepted standard in the preclinical research environment. Investigators must find an acceptable image quality (e.g., SNR, spatial resolution) that can be achieved with the lowest possible dose for each experiment. Specific experimental variables should be considered when designing any μCT study including (but not necessarily limited to) the following:

- Animal strain: Some strains have a higher radiosensitivity. Given that some animal models require specific mice strains and/or necessitate the use of immune deficient animals, any concerns specific to the strain should be tracked during the study.
- Animal gender and age: The biological response to radiation-induced damage is mediated by molecular pathways. There is experimental evidence of age specific responses to ionizing radiation, so it is worth considering both in study design.
- Tissue of interest: Local absorbed doses can vary significantly from tissue to tissue. While a particular dose may not necessarily affect one tissue (e.g., lung), it may have an impact on other tissues that absorb more local dose (e.g., bone) and both tissue types are usually exposed to radiation during a scan.
- Imaging timepoints: Some radiation-induced damage can be repaired at the molecular level. Therefore, the frequency of imaging should be considered carefully and minimized as much as possible within the context of a particular experimental hypothesis.
- Outcome metric: Some purely anatomical measurements may be less correlated to radiation-induced damage. However, damage may still be occurring at the cellular and molecular levels. This may be acceptable in some studies, but should be considered carefully particularly when a molecular-based pathology is being studied and/or when subsequent research will require investigating cellular and molecular mechanisms. This should also be considered carefully when pathologies which are inherently systemic (e.g., metastatic cancer) are being investigated.

## Conclusions

In conclusion, μCT imaging techniques are invaluable in many research studies and have been widely used. The Quantum GX imaging system has a beam quality comparable to other μCT imaging systems and provides range of protocols which enable a wide range of possible radiation doses. Selecting the best imaging protocol is not trivial. Doses can be chosen which are quite low to avoid any radiation damage, but some applications will require higher doses. There is some conflicting data in specific studies, but the complex downstream effects of radiation-induced damage make it important carefully manage radiation doses when using these techniques. Simple metrics like body weight are important to track and may uncover some underlying systemic changes when frequent longitudinal imaging is required. Including additional experimental groups with a significantly reduced radiation exposure can also provide some assurance that the radiation dose effects do not adversely impact interpretation of the data. When all of these considerations are properly balanced, μCT-based imaging techniques can be properly managed to provide all the benefits of *in vivo* imaging.
